# A robust energy management strategy for fuel cell and ultracapacitor hybrid electric vehicles under uncertainty via a jellyfish-search-based approach

**DOI:** 10.1038/s41598-025-22243-4

**Published:** 2025-10-27

**Authors:** Yara A. Morsi, Kh. M. Hasaneen, Naser Abdel-Rahim, Islam M. Abdelqawee

**Affiliations:** Department of Electrical Engineering, Faculty of Engineering at Shoubra, Benha University, Cairo, Egypt

**Keywords:** Electric vehicles, Fuel cells, Ultracapacitor, Energy management, Jellyfish search, Robust optimization, Energy science and technology, Engineering

## Abstract

This paper presents the development of an energy management system (EMS) for a fuel cell hybrid electric vehicle comprising a fuel cell (FC) and an ultracapacitor (UC). In previous studies, hydrogen consumption was the key priority, overlooking other considerations such as lifetime and characteristics of both the FC and the UC. Due to this and the restricted number of iterations, optimization strategies reported in the literature may suffer from suboptimal solutions. In addition, ignoring practical operating scenarios, such as uncertainties due to road conditions, operating status, temperature, and aging, results in poor performance. This work proposes EMS, which considers: (1) fuel usage, (2) lifespan, and the slow dynamic response of the FC, and (3) the lifetime of the UC. Because of its fast and less convergence time characteristics, the Jellyfish Search (JS) optimizer is used. To achieve resilient performance under uncertainties, robust optimization-based EMS using min-max optimization and JS is adopted. The developed EMS was tested under two different driving cycles. Based on the simulation results, the proposed EMS shows good performance. JS has a short computational time of about 0.15 s for each decision while satisfying all the system constraints, such as keeping the SoC within a suitable level (25% to 95%), and reducing the occurrences of severe changes in the power demand of fuel cells, thus increasing the life span of system components. Moreover, by adding robust optimization (RO), the system was able to meet the system requirement with DS 100% even under uncertainties.

## Introduction

Motivated by the growing pollution and the rapid depletion of fossil fuel resources, exploring new alternative sources of energy is no longer a luxury^1^. Renewable and green energy production sources like photovoltaic, wind power, hydropower, and fuel cells have gained huge attention as promising electricity generation types^2^. Developing clean transportation technologies is equally as crucial as improving electric generation, because vehicles that rely on internal combustion (ICE) engines are one of the main causes of the persistent problem of air pollution^3,4^. Thus, electric vehicles (EVs) have been adopted to limit emissions that were produced by ICE^5,6^.

Battery electric vehicles (BEVs) were developed as low-emission vehicles, but they suffer from long charging time, heavy weight, pollution during the manufacturing process, and difficulty of recycling. For these reasons, fuel cell electric vehicles (FCEVs) appear as a valuable alternative to the BEVs^7^. Fuel Cells (FCs) are considered electrochemical systems that produce electricity directly from chemical energy^8^. Hydrogen is the fuel of these cells which is eco-friendly and has nearly zero greenhouse gas (GHG) emissions^9^. Hydrogen has numerous advantages but one of the most attractive merits is the high energy density of about 122 MJ/kg (33.9 kWh/kg) which is superior to the specific energy density of fossil fuels and the gravimetric energy storage capacities of batteries^10–12^.

Despite the preceding advantages, fuel cells have certain restrictions. During the driving cycle, vehicle demand changes often; therefore, the FCs must alter their output power quickly and over a broad range. These harsh environments can accelerate the aging process^13,14^. Furthermore, the car generates regenerative power during braking or negative slopes, and fuel cells cannot use this power^15^. So, an energy storage system (ESS), as ultracapacitors (UCs), is used to obtain a smooth and effective operation of FCEV^16^. UC has the ability to provide a significant burst of demand power and has a higher power density than batteries^17^. Also, ultracapacitor can store the energy provided by the vehicle or even the fuel cell itself^18^.

**Fig. 1:**
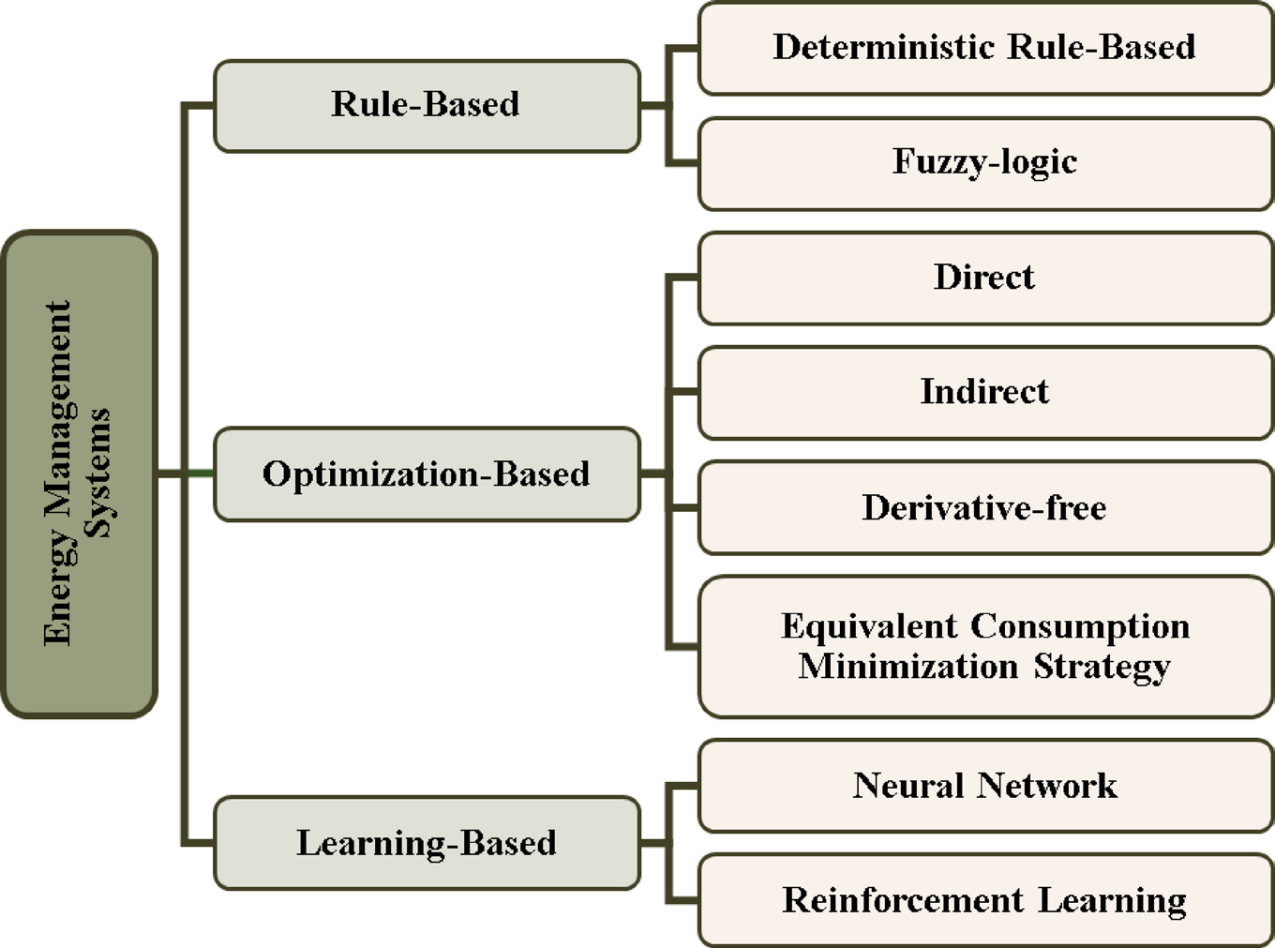
Energy management system classification.

Fuel cell hybrid electric vehicles (FCHEVs) have been developed including fuel cells system as a main power source in addition to ultracapacitor as an energy storage system. In this hybrid system, a suitable energy management system (EMS) is required for allocating and controlling the flow of power of each element within the system^19^. EMS is developed to fulfill particular aims with the main objective of meeting the power demand by vehicle^20^. Figure 1 illustrates the classification of EMSs. Recently, a lot of EMS have been developed, such as 1- rule-based; 2- optimization-based and 3- learning-based.

Rule-based EMS is the easiest and most widely used in the commercial field. This management system is built relying on mathematical models and experimental results. It is classified into two main methods deterministic rule-based method and the Fuzzy rule-based method^21^. The deterministic rule-based strategy is designed based on rules that are aided by data about fuel economy. The implementation of this method is done using lookup tables^22^. Power follower control-based EMS (PFC) and thermostat control strategy-based EMS (TCS) are the most applied deterministic rule-based EMS. A hybrid EMS that combines the deterministic rule-based and equivalent consumption minimization strategy for FCHEV, including FC/UC, is implemented in^23^, which allows the FCs to operate more efficiently and reduce hydrogen consumption. In^24^, a state machine strategy (SMS) method is developed based on droop control, which provides stable operation. The deterministic rule-based method is characterized by a simple design, which makes it the most feasible strategy of EMS. Despite its merits, this EMS does not guarantee the optimal solution.

Fuzzy logic control (FLC) is a popular control system implemented according to membership functions and IF-THEN rules^21^. In^25^ fuzzy logic strategy is used in hybrid fuel cell/battery vehicles to satisfy the power demand. Hybrid EMS includes a fuzzy logic controller, and an adaptive controller is used in^26^, where fuzzy logic is used in improving the performance adaptive controller. FLC can be implemented easily and has good robustness; however, the rules of the fuzzy controller interference must be set by experts, which makes it difficult to design^27^.

Optimization-based EMS are used to find the optimal solution that ensures the minimization of the cost function which is commonly the hydrogen consumption. This strategy is classified into direct, indirect, derivative-free free and equivalent consumption minimization strategy. Dynamic programming (DP) is an important method for global direct optimization, which uses multi-step decision-making process^21,28^. An improved dynamic programming method is used in^29^, it aims to reduce hydrogen usage considering operational cost. In^30^, a unified dynamic programming model that has four state variables is used. Because of the computational complexity of this process, DP is limited to offline optimization. Pontryagin’s minimum principle (PMP) is considered an indirect optimization-based EMS that provides near-optimal solutions. It is faster compared to dynamic programming but includes more complex mathematics. To reduce the complex mathematics of the PMP, Equivalent Consumption Minimization Strategy (ECMS) was implemented^31^.

Equivalent Consumption Minimization Strategy (ECMS) is an online optimization-based EMS that provides local optimal solutions. The main idea behind ECMS is to reduce the summation of both fuel consumption and the equivalent hydrogen consumption of auxiliary sources^31^^32^. provides an adaptive energy management system relying on ECMS, which is superior to rule-based methods and even the original ECMS itself in reducing hydrogen consumption, but it adopts ideal and simplified conditions. An equivalent consumption minimization strategy was adopted in^33^, which aims to improve the efficiency of the fuel cells system and minimize the hydrogen consumption by distributing the low-frequency component of the demand power to fuel cell system and battery modules while keeping the high-frequency component to the ultracapacitor, which can handle these rapid fluctuations in power.

DFEMS is based on meta-heuristic optimization algorithms like genetic algorithm (GA), Quantum Butterfly Optimization Algorithm (QBOA) and particle swarm optimization (PSO). In^34^, DFEMS was developed based on particle swarm optimization. DFEMS was used in both fuel cell/ultracapacitor, and fuel cell/battery hybrid electric vehicles, aiming to minimize the fuel consumption and hence reduce operation cost. This DFEMS can provide a global optimal solution, but if the number of iterations is limited, the solution could be suboptimal^35^.

Learning Based EMS (LBEMS) is built based on a huge amount of data sets, either in real-time or from historical data^36^. LB is classified into two main methods: Artificial Neural Network (ANN) and Reinforcement Learning (RL)^31^. ANN was developed based on the neurons present in the human brain. It can provide adaptability but requires a large amount of data for training^34,36^. Another type of LBEMS is the reinforcement learning-based energy management system (RLEMS)^37^. RLEMS focused on improving the fuel economy and on reducing the computational burden. The RLEMS relies on the real interactivity of the system in real life rather than collecting large historical data. RLEMS does not need any physical modeling; however, it is difficult to implement as it requires a long time for training, and there is an absence of hardware validation^31^.

Most of the work reported for FCHEV focused on one objective rather than multi-objectives. Also, earlier studies assumed ideal operating scenarios, neglecting any uncertainties that could occur in real life due to the surrounding environment, operational conditions, and aging of the equipment. The presence of these uncertainties may lead to failure in the performance of the vehicle, or at best, suboptimal operation. In this work, a robust energy management system is implemented to address all the issues mentioned above. The main contributions of this paper are as follows:

- Developing an optimization-based energy management system that considers more than one target using the jellyfish search optimizer.
- Investigating the sources of uncertainty and providing a robust optimization (RO) based on min-max optimization and jellyfish search to handle the uncertainty issue.

Table 1 presents a comparison between previously used EMS techniques and the proposed system. It shows the features and limitations of each system. The remaining of the paper is structured as follows: the description of each component in the FCHEV system will be found in Sect. “FCHEV modeling”. The proposed energy management system is presented in Sect. “Proposed EMS model”. The results are shown in Sect. “Results and discussion”. Section “Conclusions” provides the conclusions.

**Table 1:** Comparison of features and limitations of different strategies.

Reference	Power Sources Used	Control Strategy	Features	Limitations
^24^	FC + Battery + UC	SMS based on droop control	- Improving hydrogen consumption. - Ability to coordinate multiple sources.	- Uncertainty is not taken into consideration. - No limitation on the maximum change in FC power.
^26^	FC + Battery or UC	FLC strategy	- More efficient system. - Improving the battery lifetime.	- High complexity. - No consideration for FC lifespan. - It does not guarantee the minimum hydrogen consumption.
^29^	FC + Battery	DP strategy	- Minimizing hydrogen usage. - Considering the life-cycle cost.	- Uncertainty is not taken into consideration. - Better to be used offline.
^34^	FC + Battery	ECMS	- accommodating various working conditions - Minimizing hydrogen consumption.	- Not taking FC lifespan into consideration.
Proposed	FC + UC	JS Optimization-based EMS + RO	- Considering the lifespan of both FC and UC. - Providing a Robust system. - Minimizing hydrogen consumption.	- Cost of robustness.

## Fuel cell hybrid electric vehicle modeling

**Fig. 2:**
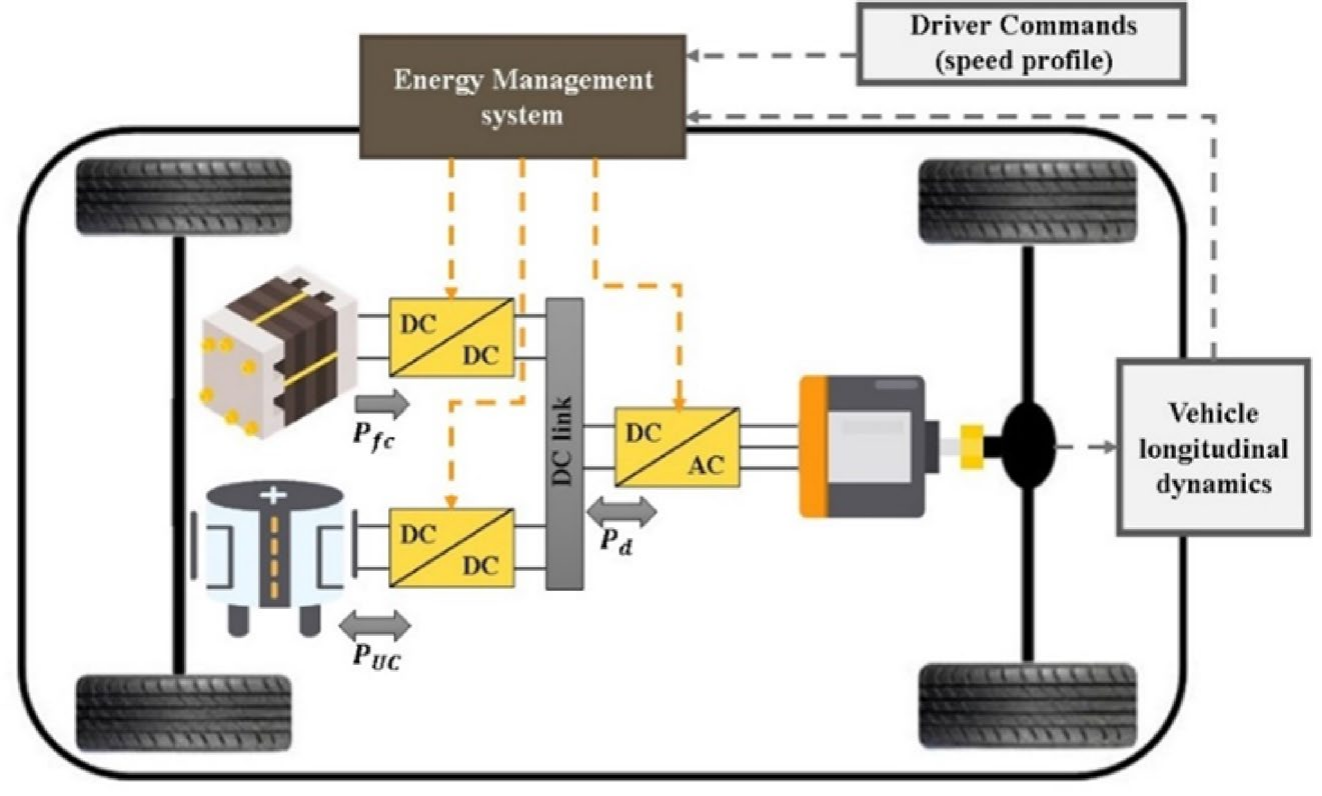
FCHEV configuration.

Figure 2 illustrates the overall system of the FCHEV. The FCHEV implemented in this paper uses an energy source (which is Fuel Cell) aiming to supply the power demanded at the motor-wheels and an energy storage system represented by (ultracapacitor). DC/DC converters are used to tie these sources with the DC link. Inverter is used to convert the DC power of the sources to AC power required by the motors. Finally, the motor is responsible for converting the electrical power to the mechanical power demanded by the vehicle. To ensure high performance of the system with acceptable efficiency level, energy management system is adopted. EMS can control the power flow from/to each source using power electronic converters. A brief modeling of each component in the system will be provided in the following subsections.

### Vehicle modeling

For estimating the vehicle power demand, the vehicle is regarded as a solid object traveling while facing forces over the longitudinal axis. A dynamic model is formulated founded on the fundamental law of mechanics, where vehicle power ($\:{P}_{v}$) is the sum of the inertia power ($\:{P}_{i}$), the contribution of aerodynamic resistance ($\:{P}_{a}$), friction force against the progress ($\:{P}_{r}$), and the force of gravity acting on the vehicle ($\:{P}_{g}$). The vehicle power can be obtained as following^38^.

1 $${P_v}={P_i}+{P_a}+{P_r}+{P_g}$$

2 $${P_i}=M \times V \times a$$

3 $${P_a}=\frac{1}{2}{\rho _{air}}{A_f}{C_d}{V^3}$$

4 $${P_r}={C_r}MVg\cos \alpha$$

5 $${P_g}=MVg\sin \alpha$$

Where:

*M* is the weight of the vehicle.

*V* is the speed of the vehicle.

*a* is the acceleration of the vehicle.

$\:{\rho\:}_{air}$ is the air density.

*A*_*f*_ is frontal surface area of the vehicle.

*C*_*d*_ is the drag coefficient.

*C*_*r*_ is the rolling resistance.

*g* is gravitational constant.

$\:\alpha\:$ is the road gradient.

### Fuel cells model

Proton exchange membrane fuel cell (PEMFC) employs the electrochemical reaction between oxygen and hydrogen that produces electricity with heat and water vapor as a byproduct. The model of the electrochemical reactions of the PEMFC have been developed in^39,40^ and is given by.

6 $${H_2} \to 2{H^+}+2{e^ - }$$

7 $$2{H^+}+2{e^ - }+0.5{O_2} \to {H_2}O$$

8 $${H_2}+0.5{O_2} \to {H_2}O+Energy$$

The output voltage of the FC (*V*_*FC*_) and generated emf of a one cell (*E*_*N*_) are expressed as^38^:

9 $${V_{FC}}={E_N} - {V_{Act}} - {V_{Ohm}} - {V_{Con}}$$

10 $${E_N}=1.229 - 0.85 \times {10^{ - 3}}({T_{st}} - 298.15)+4.3085 \times {10^{ - 5}}{T_{st}}(\ln ({P_{{H_2}}})+0.5\ln ({P_{{O_2}}}))$$

Where:

*V*_*Act*_ is the activation voltage drop.

*V*_*Ohm*_ is the ohmic voltage drop.

*V*_*Con*_ is the concentration voltage drop.

*T*_*st*_ is the temperature of the stack.

$\:{P}_{{H}_{2}}$ is the partial pressure of hydrogen.

$\:{P}_{{O}_{2}}$ is the partial pressure of oxygen.

The net output power produced by the fuel cells ($\:{P}_{fc})$, taking into consideration hydrogen input power ($\:{P}_{H})$ and efficiency of the whole system ($\:{\eta\:}_{fc})$, can be given by:

11 $${P_{fc}}={\eta _{fc}}{P_H}$$

Where the overall efficiency is given as.

12 $${\eta _{fc}}={\eta _a}{\eta _s}$$

Where:

$\:{\eta}_{a}$ is the auxiliary’s efficiency.

$\:{\eta}_{s}$ is the stack efficiency.

So, the hydrogen consumption $\:{C}_{{H}_{2}}$(in grams) is given by.

13 $${C_{{H_2}}}=\frac{{{P_{fc}}}}{{{\eta _{fc}}({P_{fc}}) \times LHV}}$$

Where LHV is the lower heating value of hydrogen.

### Ultracapacitor model

Due to the limited capabilities of FC to supply sudden power demand in accordance with vehicle operating conditions, ultracapacitors are used to supplement the sudden power requirement of the vehicle. This has the advantage of prolonging the lifetime of the FC. In addition, since FC is incapable of receiving regenerative energy generated by the vehicle during vehicle deceleration, ultracapacitors are used to store this regenerative energy and hence improving the overall efficiency of the EV. Ultracapacitors are energy storage systems which have superior power density and fast charging/discharging operation^41,42^.

Each single cell of ultracapacitor can be represented as an RC circuit. Based on ohmic losses (*P*_*ohm*_) and ultracapacitor raw power (*P*_*UC, raw*_) the supplied or stored power (*P*_*UC*_) can be calculated^43^.

14 $${P_{ohm}}=I_{{UC}}^{2}{R_{UC}}$$

15 $${P_{UC}}=\left\{ {\begin{array}{*{20}{c}} {\begin{array}{*{20}{c}} {{P_{UC,raw}} - {P_{ohm}}}&{\begin{array}{*{20}{c}} {{\text{In}}}&{{\text{discharge}}} \end{array}} \end{array}} \\ {\begin{array}{*{20}{c}} {{P_{UC,raw}}+{P_{ohm}}}&{\begin{array}{*{20}{c}} {{\text{In}}}&{{\text{charging}}} \end{array}} \end{array}} \end{array}} \right.$$

The *SoC* of the UC can be represented as:

16 $$SoC=\frac{{{E_{UC}}}}{{{E_{Max}}}}$$

Where:

*E*_*UC*_ is the stored energy of the UC.

*E*_*Max*_ is the maximum energy of the UC.

### Power electronic converters

In FCHEV power converters are essential parts in this system. A high-gain DC/DC converter is used for boosting and stabilizing the output voltage of the FC, and a bidirectional DC/DC converter is employed to boost voltage and allow the power to flow in both directions for charging/discharging the ultracapacitor^42^. In addition, the DC/DC converters are employed in managing the power flow between sources and load. Detailed modeling of power converters to compute its efficiency is reported in^43^. In this work, a rather simple model that considers the converter’s input $\:{(P}_{input})$ and output powers $\:{(P}_{output})$, is used:

17 $${\eta _{Conv}}=\left\{ {\begin{array}{*{20}{c}} {\frac{{{P_{cnv,\operatorname{li} nk}}}}{{{P_{cnv,source}}}}}&{If}&{{P_{cnv,\operatorname{li} nk}} \geq }&{({\text{discharging}})} \\ {\frac{{{P_{cnv,source}}}}{{{P_{cnv,\operatorname{li} nk}}}}}&{If}&{{P_{cnv,\operatorname{li} nk}}<0}&{({\text{charging}})} \end{array}} \right.$$

Where:

*P*_*cnv, source*_ is the power entering/leaving the power converter from source side.

*P*_*cnv*_,_*link*_ is the power entering/leaving the power converter from dc link side.

### Uncertainty in the system

In real life many factors could lead to a variation in nominal values of system parameters. These fluctuations will affect the performance of the system. Three levels of uncertainty are considered in this work:

#### Uncertainty in power demand

- Vehicle power demand: drag coefficient $\:\left({\text{C}}_{\text{d}}\right)$ and rolling friction coefficient $\:\left({\text{C}}_{\text{r}}\right)$ are both taken as nominal values during calculation of vehicle power $\:\left({\text{P}}_{\text{v}}\right)$. In actual scenarios, condition of the road, tire pressure and other operating conditions can cause deviation in $\:{\text{C}}_{\text{d}}$ and $\:{\text{C}}_{\text{r}}$. On this account, these variables will be included as uncertainty intervals during optimization procedure^43^.
- Motor-wheels: the overall amount required at the motor-wheels is given by the summation of losses at the motor-wheels and propulsion power. Therefore, any change in the efficiency of motor-wheels will affect the total required power. Motor-wheels efficiency $\:{({\upeta\:}}_{\text{m}})$ is influenced by aging and operational conditions, so it will also be included as uncertainty intervals instead of fixed value.

#### Uncertainty in power converter

Many studies take the efficiency of the inverter and DC/DC converter as a nominal value, however, under real conditions these values diverge from the nominal values. Since power converters efficiency and energy losses are primary concerns in optimization process, converter and inverter efficiency will be treated as uncertain intervals^44^.

#### Uncertainty in power generation

Temperature fluctuation, stress factors, severe road conditions, oxygen deficiency, pressure of gas, aging and other degradation problems surely influence the performance and parameters of the fuel cells leading to reduction of efficiency ($\:{{\upeta\:}}_{\text{f}\text{c}})$^45^. Considering the vital role of the efficiency of FC in making the optimization decision, $\:{{\upeta\:}}_{\text{f}\text{c}}\:$is regarded as uncertain interval.

### Energy management system

**Fig. 3:**
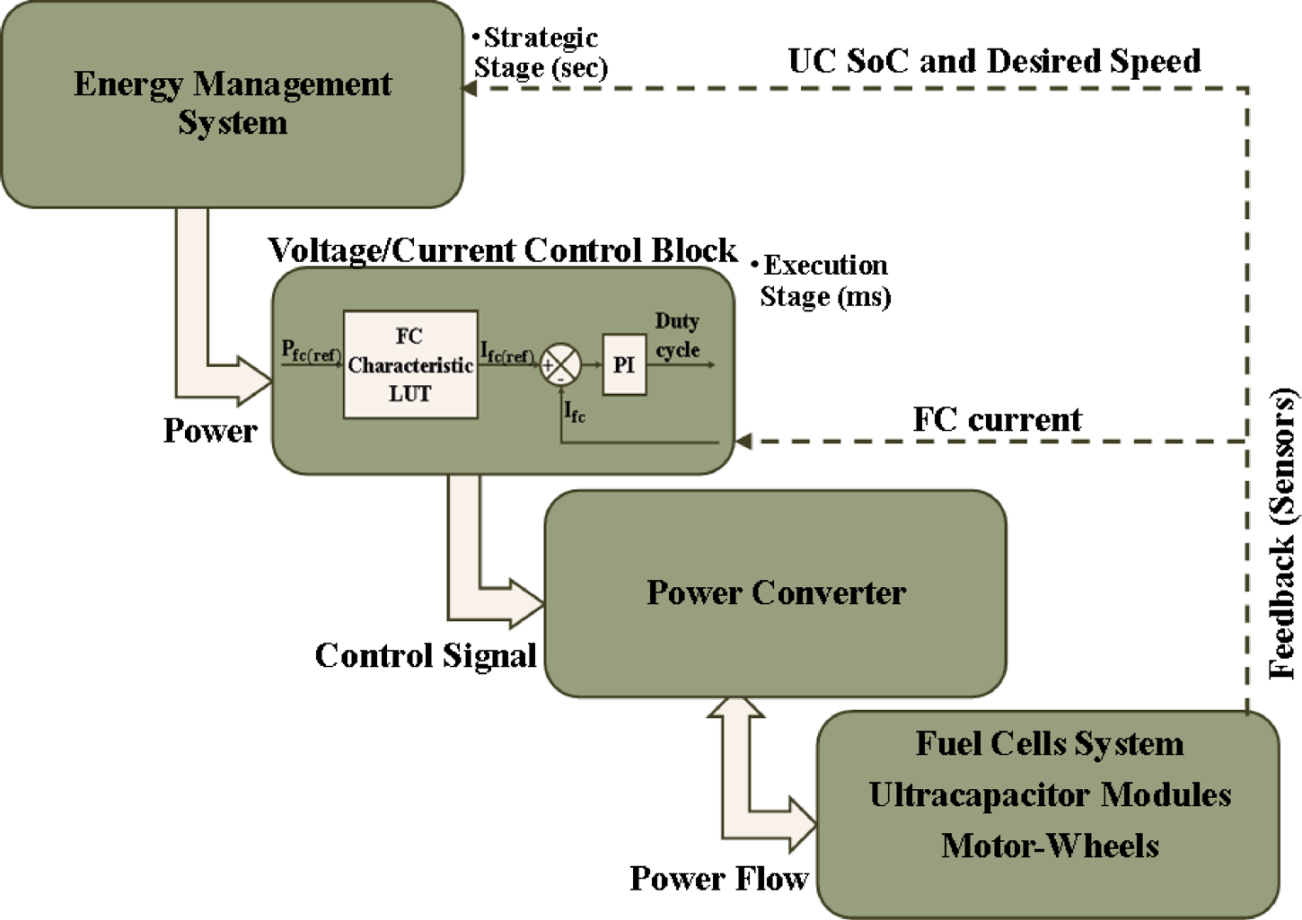
Energy control.

Utilization of UC module can overcome the limitations imposed by the FC, but it introduces a new challenge, which is how to coordinate between these two power sources. Energy management systems are developed with the aim of allocating the power shared by each energy source in the system. The core objective of the EMS is to meet the power required by the vehicle in addition to ensuring optimal performance of the system^33^. Figure 3 illustrates the control configuration of the system. EMS is considered the strategic level of the control system that provides reference values of power of each source, which are then used by the voltage/current control block for the purpose of generating the desired control signals for power electronic converters. The voltage/current control block could be any control technique, such as a lookup table (LUT) that can make use of the reference power value and turn it into the analog value of voltage/current, which is compared with the actual value of voltage/current and the error is then used by the controllers, like PI, to produce the required duty cycle for power converters. This block is not limited to this configuration only; any other technique that can provide the same aim can be used. To achieve optimal results, EMS makes use of the system restrictions, instantaneous power need P_d_ (t), state variables represented by the state of charge (SoC (t-1)) and the FC power (P_fc_ (t)), and the system variables.

## Proposed EMS model

### Certainty-based problem formulation

The Certainty-based problem formulation proposed EMS for the FCHEV seeks to minimize the hydrogen consumption of the fuel cell, while trying to keep the SoC within an acceptable level to avoid any unfeasible solutions at extremely high and low demand power. The main constraint of this problem is the instantaneous equilibrium of the power produced and power demand to ensure high performance of the vehicle. In addition to this main constraint, there are some limitations due to the design constraints of each source in the system. So, inequality constraints are made for the maximum and minimum values of power generated by fuel cells system and state of charge of ultracapacitor module. For the purpose of avoiding oxygen deficiency problem in FC system, the instant change in power produced by FC system must be kept within a suitable limit. This slow dynamic characteristic imposed by fuel cells nature is also formulated by inequality constraints. In this optimization problem, the cost function is designed to minimize the hydrogen consumption of FC system and try to keep SoC of UC intermediate as possible. The state equation here is the SoC of UC module. The Certainty-based optimization problem is articulated as follows:

18 $$\mathop {\hbox{min} }\limits_{{{P_{fc}}}} \left\{ {C(k)} \right\}=\mathop {\hbox{min} }\limits_{{{P_{fc}}}} \left\{ {{C_{{H_2}}}(k)+{C_{UC}}(k)} \right\},$$

19 $${C_{{H_2}}}(k)=\frac{{{P_{fc}}(k)}}{{{\eta _{fc}}({P_{fc}}(k)) \times LHV}},$$

20 $${C_{UC}}(k)=0.6 - SoC(k).$$

Equation (18) to (20) are subject to:

21 $$SoC(k+1)=SoC(k) - {P_{UC}}(k)\frac{{\Delta T}}{{{E_{Max}}}}$$

22 $${\eta _{Conv,fc}}{P_{fc}}(k)+{\eta _{Conv,UC}}{P_{UC}}(k) - {P_d}(k)=0$$

23 $${P_{fc,\hbox{min} }} \leq {P_{fc}}(k) \leq {P_{fc,\hbox{max} }}$$

24 $$\Delta {P_{fc,fall}}\Delta T \leq {P_{fc}}(k) - {P_{fc}}(k - 1) \leq \Delta {P_{fc,rise}}\Delta T$$

25 $$So{C_{\hbox{min} }} \leq SoC(k) \leq So{C_{\hbox{max} }}$$

Where:

*C(k)* is the objective function to be minimized of.

*C*_*H2*_ is the objective function of hydrogen consumption.

*C*_*UC*_ is the objective function of the difference between the current and the desired of the SoC.

*P*_*fc, min*_ is FC minimum power.

*P*_*fc, max*_ is FC maximum power.

*SoC*_*min*_ is the minimum SoC of UC.

*SoC*_*max*_ is the maximum SoC of UC.

*ΔP*_*fc, fall*_ is the maximum power falling rate of FC.

*ΔP*_*fc, rise*_ is the maximum power rising rate of FC.

The objective function aiming for minimization is shown in (18). Equation (21)represents the state equation that describes the variation of SoC at each step. The equality constraint provided by (22) ensures the power balance within the system, where the power demand (P_d_) is given by following equation. 

26 $${P_d}(k)=\frac{{{P_v}(k)}}{{{\eta _{inv}}{\eta _m}}}$$

Where:

$\:{\eta\:}_{inv}$ is the efficiency of the inverter.

$\:{\eta\:}_{m}$ is the efficiency of the motor.

Inequality equations (23) to (25) satisfy the limitations imposed by the sources within the system. These equations take into consideration the capacity limits of FC and UC systems, and the slow dynamic nature of the fuel cells.

### Jellyfish search algorithm

Jellyfish search is a recently developed metaheuristic optimization algorithm that was motivated by the behavior of jellyfish while searching for food in large oceans. Compared with previous algorithms, JS provides favorable and promising results by doing adequate work combining exploitation and exploration to achieve optimal values^46^. At the beginning of the movement, jellyfish are attracted to the ocean current. This behavior arises from the fact that ocean currents are rich in nutrients. The new position in this case is given by the following.

27 $${X_i}(t+1)={X_i}(t)+rand(0,1) \times \overrightarrow {trend}$$

28 $${X_i}(t+1)={X_i}(t)+rand(0,1) \times ({X^*} - \beta \times rand(0,1) \times \mu )$$

Where:

*X* is the position vector of jellyfish.

$\:\overrightarrow{trend}$ indicates the ocean current’s direction.

$\:{X}^{*}$ is the jellyfish that has the best location within the swarm.

$\:\beta\:$ is the distribution coefficient.

$\:\mu\:$ is the mean position of all jellyfish.

$\:{X}^{*}$ is the jellyfish that has the best location within the swarm.

Over time, jellyfish start to move in swarms in both passive (type A) and active (type B) motions. As the swarm was initially created, most jellyfish exhibit passive motion. In this type of motion, jellyfish move around their own positions, and the corresponding position updated is given by.

29 $${X_i}(t+1)={X_i}(t)+\gamma \times rand(0,1) \times ({U_b} - {L_b})$$

Where:

*L*_*b*_ is the lower bound of search space.

*U*_*b*_ is the upper bound of search space.

$\:\gamma\:$ is the motion coefficient.

As time goes on, a greater number of jellyfish start to exhibit active motion. In active motion, a random jellyfish (j) beside the desired jellyfish (i) are selected. The vector from jellyfish (i) to jellyfish (j) is employed to find the direction of movement. The jellyfish(i) move either toward or away from the position of jellyfish (j) based on whether the amount of food at jellyfish (j) position is more or less than that at jellyfish (i). This is done as follows.

30 $$\overrightarrow {Step} =rand(0,1) \times \overrightarrow {Direction}$$

31 $$\overrightarrow {Direction} =\left\{ {\begin{array}{*{20}{c}} {\begin{array}{*{20}{c}} {{X_j}(t) - {X_i}(t)}&{if}&{f({X_i}) \geqslant f({X_j})} \end{array}} \\ {\begin{array}{*{20}{c}} {{X_i}(t) - {X_j}(t)}&{if}&{f({X_i})<f({X_j})} \end{array}} \end{array}} \right.$$

32 $${X_i}(t+1)={X_i}(t)+\overrightarrow {Step}$$

To find the type of motion as time passes, a time control mechanism is used. It regulates not just whether the motion is type A and type B in swarms, but also the movements of jellyfish toward the ocean current. The time control function *c(t)* is given as.

33 $$c(t)=\left| {\left( {1 - \frac{t}{{Ite{r_{\hbox{max} }}}}} \right) \times \left( {2 \times rand(0,1) - 1} \right)} \right|$$

Where:

*Iter*_*max*_ is the maximum number of iterations.

**Fig. 4:**
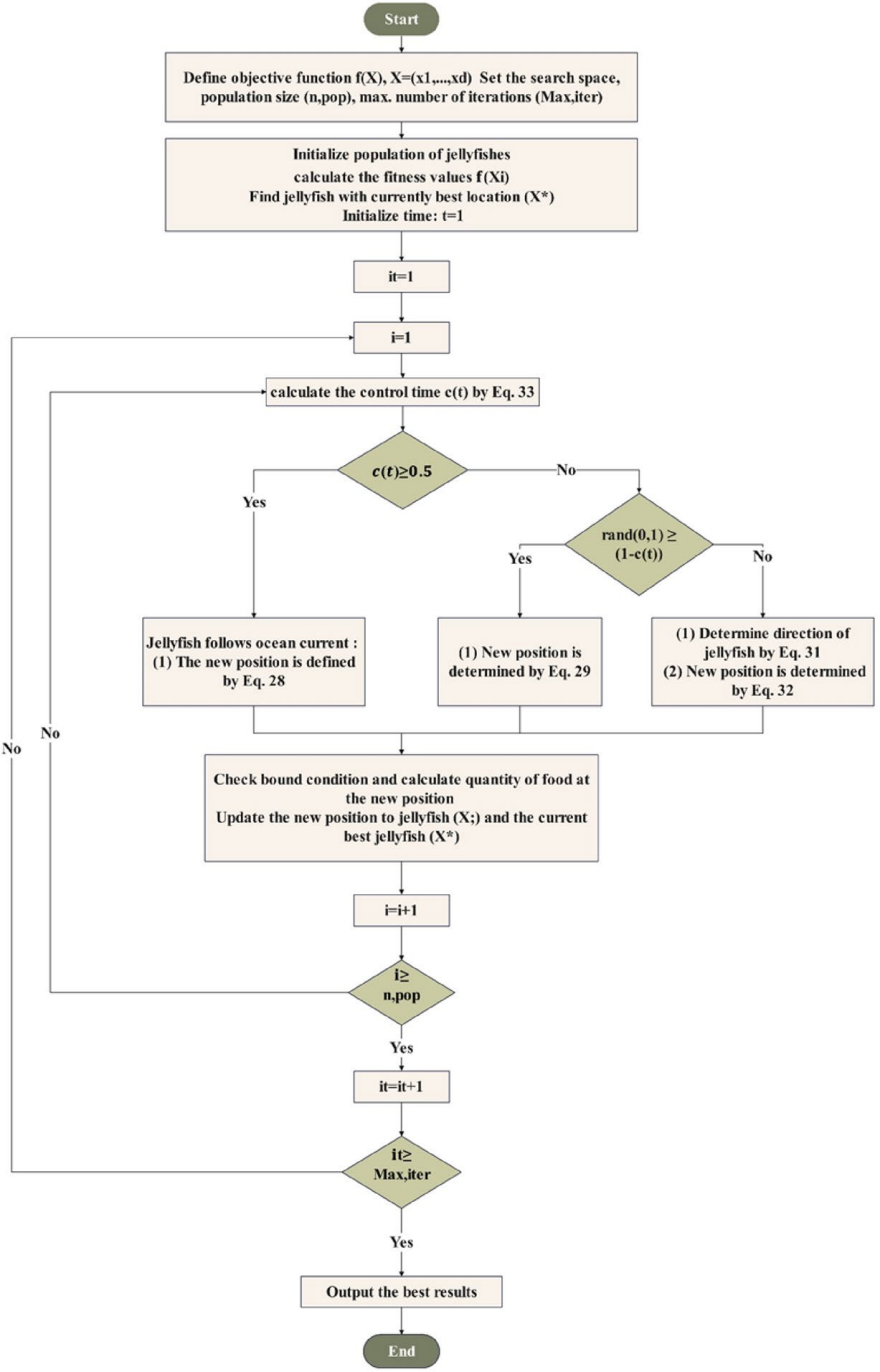
Flow chart of jellyfish search optimizer.

The JS is characterized by faster and less time convergence. It gets the best results while balancing between exploration and exploitation, owing to a time control mechanism that allows switching the movement^47^. Due to these properties, JS is used in solving the EMS problem in FCHEV, and the flowchart is illustrated in Fig. 4.

**Fig. 5:**
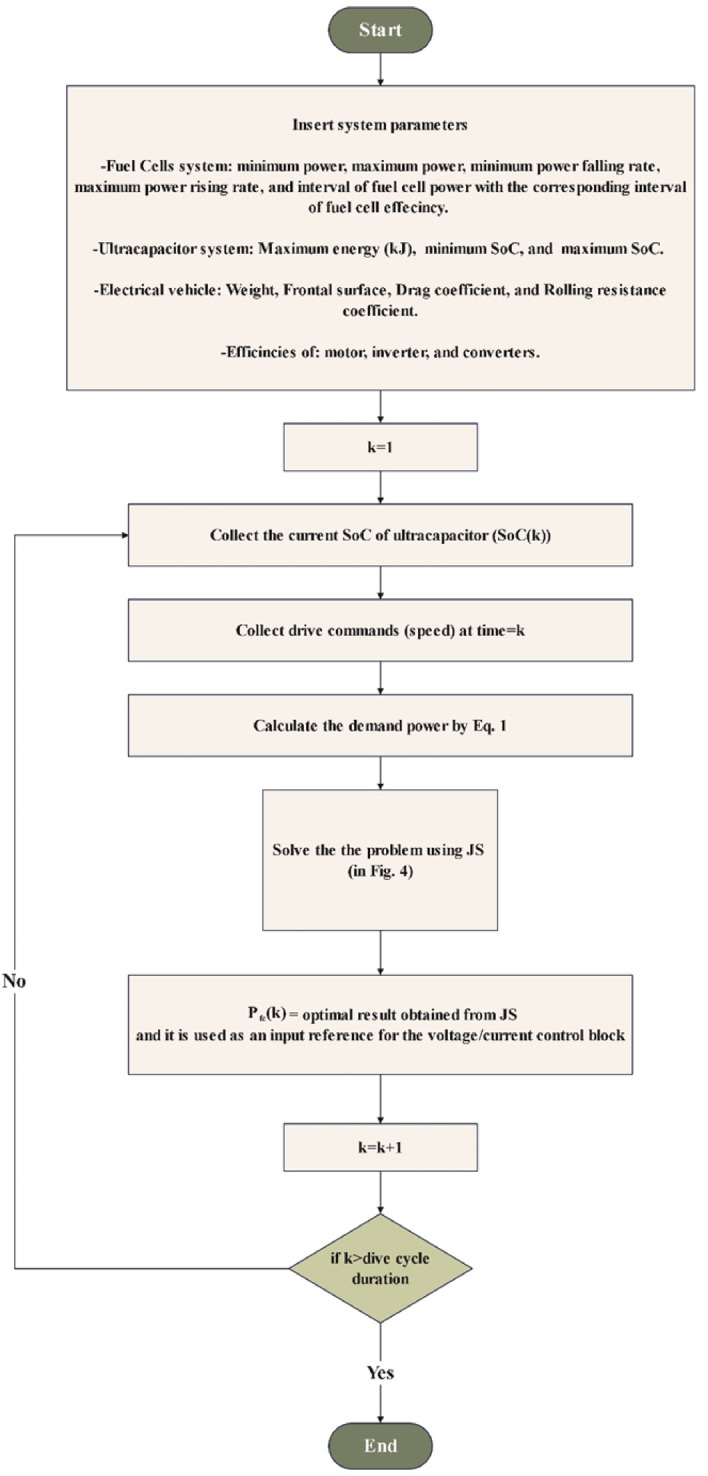
Proposed energy management system flowchart.

The flowchart of the proposed energy management system is shown in Fig. 5. At each sample time, the previous value of the FC power and the current value SoC of the UC are collected. Also, the speed command required by the driver is taken and turned into the value of the desired power demand. All these values enter the jellyfish optimizer, which provides the optimal value to minimize the previously mentioned cost function. The jellyfish position is the power of fuel cells, and the maximum amount of food is analogous to the minimum value of cost function. The optimal value produced by the optimizer is the optimal value of FC power at this time. This value is used by the voltage/current block to provide a suitable control signal for the power converters. Also, the value is memorized to be used at the next sample time as the new previous value of the FC power.

### Robust optimization model

A robust optimization technique is developed in this section to account for the uncertainties occurring during the real time operation. In this technique, the uncertain parameters are described by a set with all possibilities instead of fixed values. The solution to the optimization problem is done considering the worst-case scenario. Using the worst-case solution ensures the high performance of the FCHEV even under perturbations. In order to solve this type of problem, the min-max robust optimization introduced by Bertsimaset et al. is utilized^48^. This RO can incorporate uncertainties in not only the cost function but also constraints. The uncertainties are defined by their nominal and uncertainty intervals. So, the vector of the uncertain values is represented by the following.

34 $${\tilde {P}_{un}}(i)={P_{un}}(i)+\alpha (i);\alpha (i) \in \Delta i$$

35 $${\tilde {P}_{un}}(i)=\{ \mathop {{C_d}}\limits^{\sim } ,\mathop {{C_r}}\limits^{\sim } ,\mathop {{\eta _m}}\limits^{\sim } ,\mathop {{\eta _{conv}}}\limits^{\sim } ,\mathop {{\eta _{fc}}\} }\limits^{\sim }$$

36 $${P_{uc}}(i)=\{ {C_d},{C_r},{\eta _m},{\eta _{conv}},{\eta _{fc}}\}$$

37 $$\Delta i=\left\{ {{{\tilde {P}}_{un}}(i);\left| {{{\tilde {P}}_{un}}(i) - {P_{un}}(i)} \right|=\left| {\alpha (i)} \right| \leqslant {\alpha ^m}(i)} \right\}$$

Where:

$\:{\stackrel{\sim}{P}}_{un}$ is the vector of uncertainties.

$\:{P}_{un}$ is the vector of nominal values.

$\:\alpha\:\left(i\right)$ is the uncertainty of $\:{\text{i}}^{\text{t}\text{h}}$ element in $\:{\stackrel{\sim}{P}}_{un}$.

$\:\varDelta\:i$ is the uncertainty interval.

$\:{\alpha\:}^{m}\left(i\right)\:$ is the maximum value of the uncertainty $\:{\upalpha\:}\left(\text{i}\right)$.

So, the uncertainty interval can be given as $\:\left[{P}_{un}\left(i\right)-{\alpha\:}^{m}\left(i\right),{P}_{un}\left(i\right)+{\alpha\:}^{m}\left(i\right)\right]$. After including the uncertainty intervals in the main optimization problem by applying the min-max robust optimization, the problem can be formulated as follows.

38 $$\mathop {\hbox{min} }\limits_{{{P_{fc}}}} \left\{ {C(k,\alpha )} \right\}=\mathop {\hbox{min} }\limits_{{{P_{fc}}}} \left\{ {\mathop {\hbox{max} }\limits_{\alpha } ({C_{{H_2}}}(k,\alpha )+{C_{UC}}(k,\alpha ))} \right\},$$

39 $${C_{{H_2}}}(k,\alpha )=\frac{{{P_{fc}}(k)}}{{\left( {{\eta _{fc}}({P_{fc}}(k))+{\alpha _{{\eta _{fc}}}}} \right) \times LHV}},$$

40 $${C_{UC}}(k,\alpha )=(0.6 - SoC(k)).$$

Subjected to:

41 $$SoC(k+1)=SoC(k) - {P_{UC}}(k)\frac{{\Delta T}}{{{E_{Max}}}}$$

42 $$({\eta _{Conv,fc}}+{\alpha _{{\eta _{conv,fc}}}}){P_{fc}}(k)+({\eta _{Conv,UC}}+{\alpha _{{\eta _{Conv,UC}}}}){P_{UC}}(k) - {P_d}(k)=0$$

43 $${P_{fc,\hbox{min} }} \leq {P_{fc}}(k) \leq {P_{fc,\hbox{max} }}$$

44 $$\Delta {P_{fc,fall}}\Delta T \leq {P_{fc}}(k) - {P_{fc}}(k - 1) \leq \Delta {P_{fc,rise}}\Delta T$$

45 $$So{C_{\hbox{max} }} \leq SoC(k) \leq So{C_{\hbox{min} }}$$

Where the perturbed demand power ($\:{\stackrel{\sim}{P}}_{d})$ is be determined as following.

46 $${\tilde {P}_d}(k)=\frac{{{{\tilde {P}}_v}(k)}}{{({\eta _{inv}}+{\alpha _{{\eta _{inv}}}})({\eta _m}+{\alpha _{{\eta _m}}})}}$$

47 $${\tilde {P}_v}={\tilde {P}_a}+{\tilde {P}_r}+{P_g}+{P_i}$$

48 $${\tilde {P}_a}=\frac{1}{2}{\rho _{air}}{A_f}({C_d}+{\alpha _{{C_d}}})V_{v}^{3}$$

49 $${\tilde {P}_r}=({C_r}+{\alpha _{{C_r}}}){M_v}{V_v}g\cos \alpha$$

## Results and discussion

Table 2 shows the parameters of the (FCHEV) system and Table 3 shows the nominal values of the parameters subject to uncertainties along with its uncertainty intervals. The proposed EMS is used on FCHEV to minimize the hydrogen consumption of the FC system while maintaining the system constraint. The MATLAB platform is used for the procedure.

**Table 2:** Parameters of the system^44^.

System	Parameters	Value
Fuel Cells	Minimum power (kW)	0
	Maximum net power (kW)	20
	Minimum power falling rate (kW/s)	−3
	Maximum power rising rate (kW/s)	1.1
Ultracapacitors	Unit cell capacitance (F)	2600
	Serial number of cells	22
	Parallel number of cells	5
	Maximum energy (kJ)	1890
	$\:{\text{S}\text{o}\text{C}}_{\text{m}\text{i}\text{n}}$	0.25
	$\:{\text{S}\text{o}\text{C}}_{\text{m}\text{a}\text{x}}$	0.95
Electric Vehicle	Weight (kg)	700
	Frontal surface ($\:{\text{m}}^{2}$)	2.59
	Drag coefficient	0.3
	Rolling resistance coefficient	0.012

**Table 3:** Nominal values and uncertainty ranges for parameters^49,50^.

Parameters	Nominal value	Uncertainty interval
Drag coefficient $\:{\text{C}}_{\text{d}}$	0.3	[0, 3%]
Rolling resistance $\:{\text{C}}_{\text{r}}$	0.012	[0, 3%]
Motor wheel efficiency	0.95	[0, 2%]
Power converters efficiency	0.97	[0, 2%]
FC system efficiency	-	[0, 2%]

### Evaluation criteria

Because the primary focus of the study is on EMS, confirming the power balance between the power demanded and the power generated by various sources is critical to system performance. Surplus or deficit in providing the required power will result in system failure, hence the demand satisfaction indicator (*DS*) is employed to evaluate system performance. It is defined as the following expression^43^:

50 $$DS=100\left[ {1 - \frac{{\left| {{P_d} - {P_p}} \right|}}{{{P_d}}}} \right]$$

Where:

*P*_*d*_ is the power demand.

*P*_*p*_ is the total power supplied by system sources.

Given the possible uncertainties, the robust solution predicts a higher cost than the deterministic study. This rise is represented by the cost of robustness (*CR*), which is given by the following equation^43^:

51 $$CR=100\left[ {\frac{{{C_{robust}} - {C_{\det }}}}{{{C_{\det }}}}} \right]$$

Where:

*C*_*robust*_ is the cost of the robust solution.

*C*_*det*_ is the cost of the deterministic solution.

### Driving cycles

**Fig. 6:**
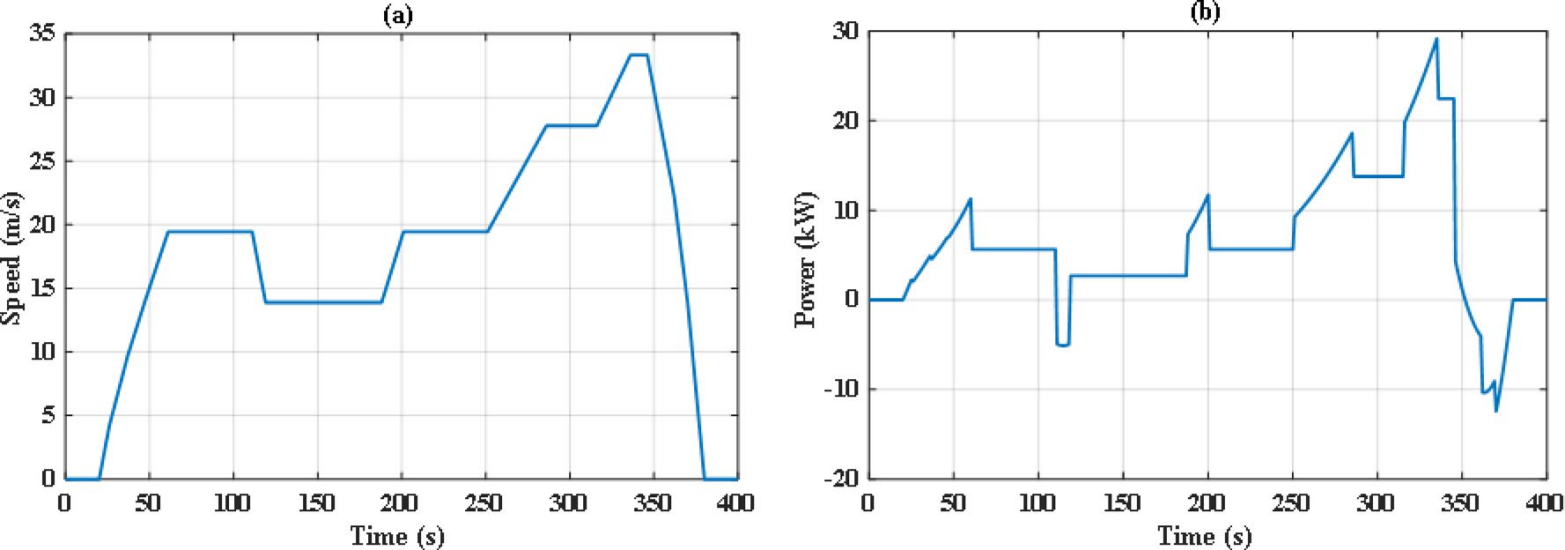
Speed and power demand of the Extra-Urban Driving Cycle (EUDC).

**Fig. 7:**
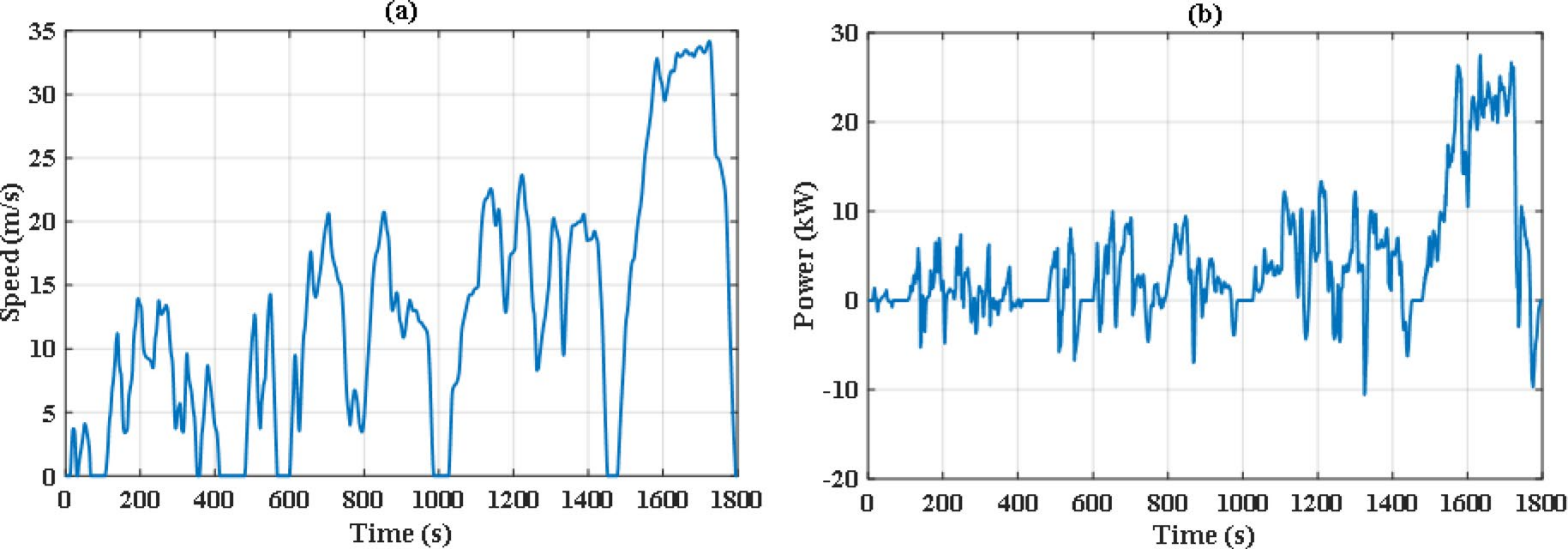
Speed and power demand of the Worldwide harmonized Light vehicles Test Cycle (WLTC-Class 2).

The simulation includes two different drive cycles: the Extra-Urban Driving Cycle (EUDC), and the Worldwide harmonized Light vehicles Test Cycle (WLTC-Class 2) as depicted in Figs. 6 and 7^51^. EUDC is a 400 s drive cycle that is featured by rapid acceleration and high speed with maximum speed of 33.33 m/s. On the other hand, WLTC-Class 2 is longer and includes four phases of speed: low, medium, high, and extra high. The idea behind choosing these drive cycles is their distinct dynamic nature and duration, which will provide better evaluation of the proposed energy management system under different driving conditions. The proposed EMS is tested using the PSO and JS algorithm under nominal conditions. Later on, the robust scenario is investigated for the JS algorithms.

### Deterministic optimization approach

#### PSO algorithm

**Fig. 8:**
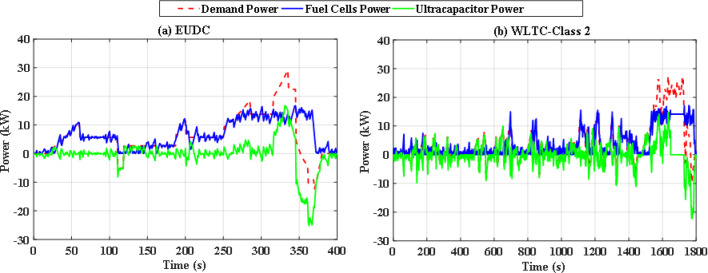
PSO-based optimization power distribution for **(a)** EUDC and **(b)** WLTC-Class 2.

**Fig. 9:**
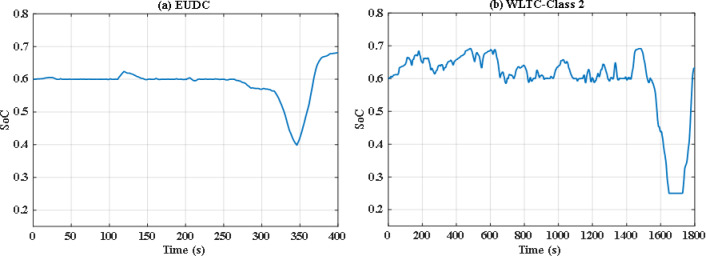
PSO-based optimization SoC of the ultracapacitor for **(a)** EUDC and **(b)** WLTC-Class 2.

**Fig. 10:**
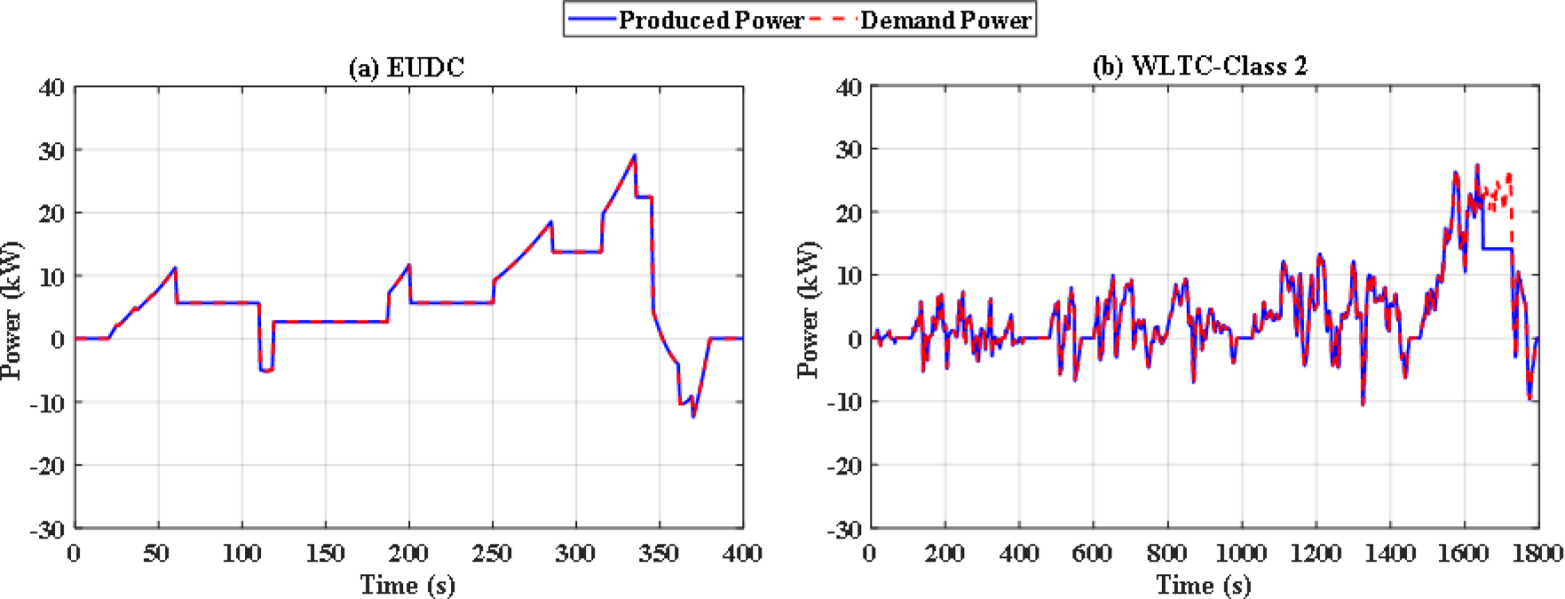
PSO-based optimization demand power and produced power for **(a)** EUDC and **(b)** WLTC-Class 2.

In this situation, all uncertainties are ignored, and the system is assumed to function at nominal levels. Two optimization techniques are utilized with the same iterations and population size to keep the computation time to an acceptable level. Particle swarm optimization (PSO), a popular optimization method previously utilized in FCHEV’s EMS, is used in this system. Figure 8(a) and Fig. 8 (b) demonstrate power distribution of EUDC and WLTC-Class 2 drive cycles respectively, while Fig. 9(a) and Fig. 9(b) show the UC’s state of charge for both drive cycles. Figure 8(a) and Fig. 8(b) show that the PSO failed to provide the optimal solution for the problem in both drive cycles with this restricted number of iterations, resulting in unwanted oscillations in FC power, and even the SoC tends to fall below 25% in WLTC-Class 2 indicating that the system will not be able to provide the demand power. Figure 10(a) and Fig. 10(b) shows the difference between the demand and produced power for both EUDC and WLTC-Class 2, as observed from this figure the two power curves are not the same in case of WLTC-Class 2, which indicates that there is deficit in produced power, and the DS of this situation is 90.8%.

#### Jellyfish search algorithm

**Fig. 11:**
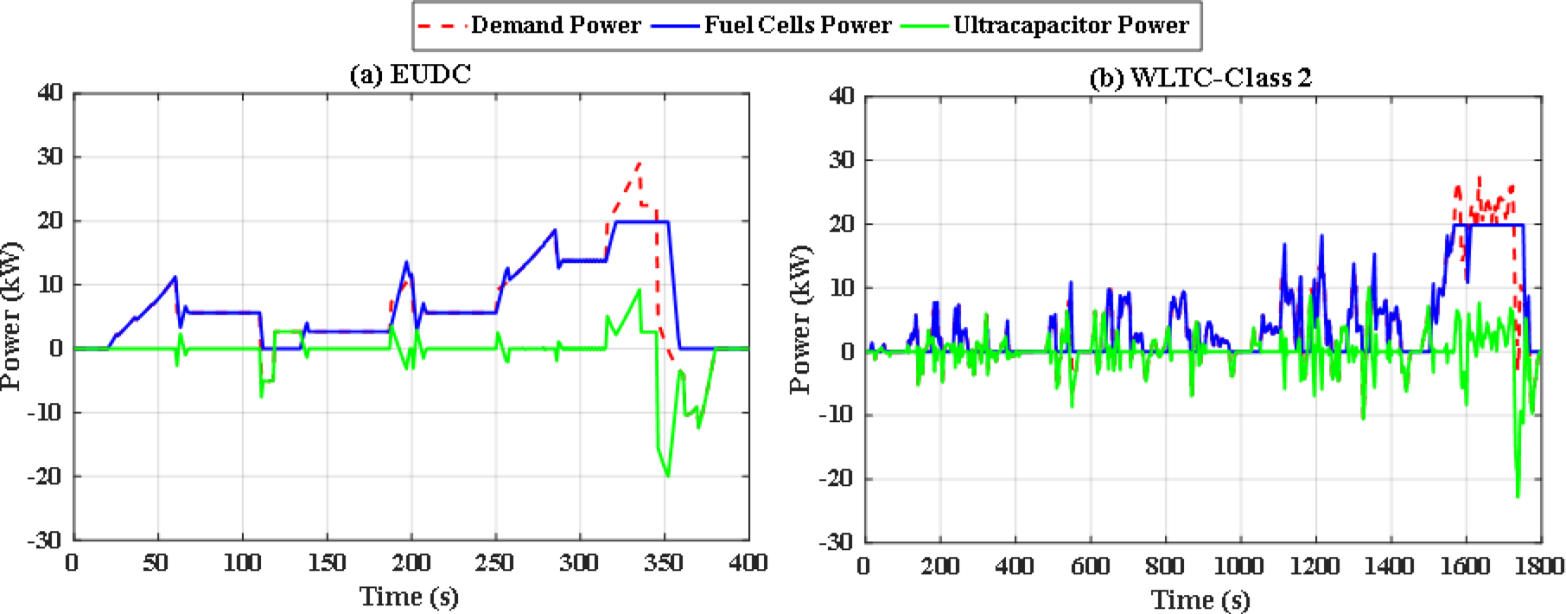
JS-based optimization power distribution for **(a)** EUDC and **(b)** WLTC-Class 2.

**Fig. 12:**
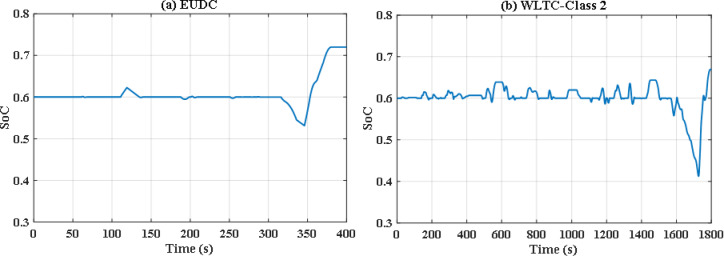
JS-based optimization SoC of the ultracapacitor for **(a)** EUDC and **(b)** WLTC-Class 2.

**Fig. 13:**
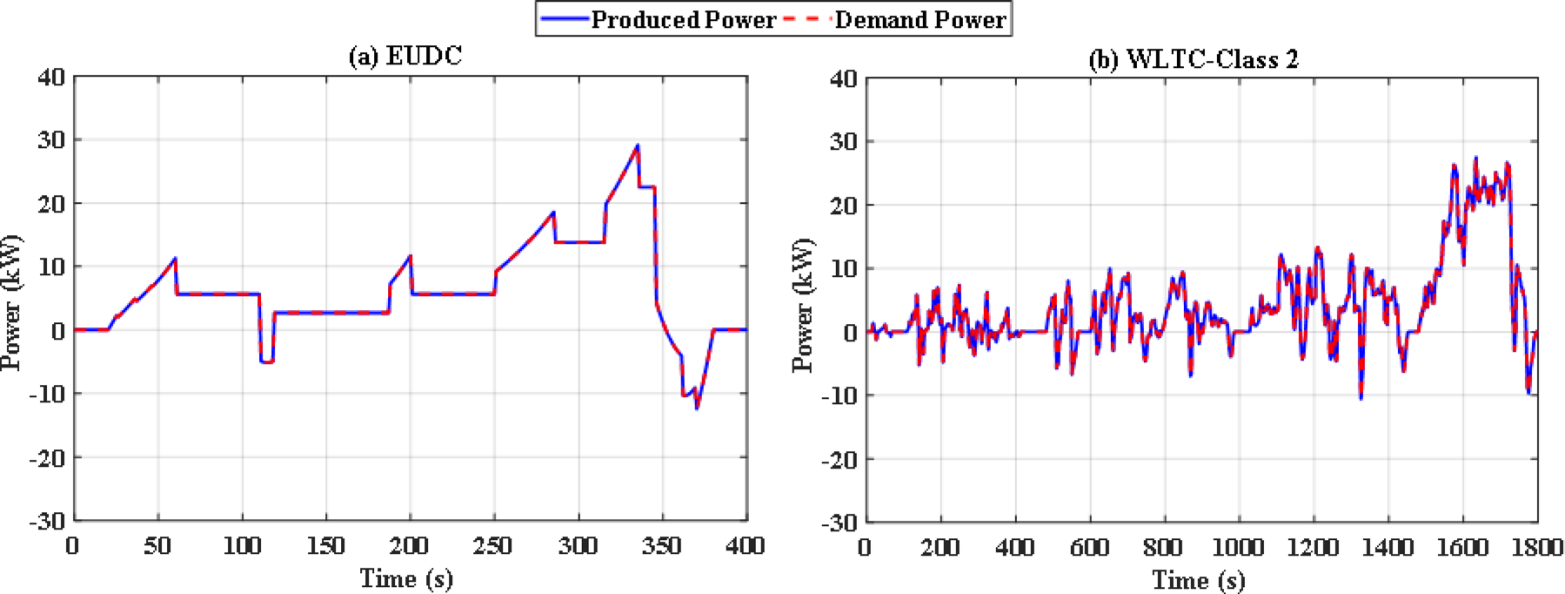
JS-based optimization demand power and produced power for **(a)** EUDC and **(b)** WLTC-Class 2.

To overcome the shortcoming of the PS algorithm, jellyfish search optimizer (JS) is investigated. The results with JS optimizer are displayed in Fig. 11(a) and Fig. 11(b). The figures show the power distribution for EUDC and WLTC-Class 2 of JS-based EMS, respectively, while Fig. 12(a) and Fig. 12(b) indicate the state of charge of their UCs. Figure 13(a) and Fig. 13(b) show the difference between the demand and produced power for both EUDC and WLTC-Class 2. As can be observed, the JS performance outperforms that of the PSO because there are no unwanted oscillations and the SoC is kept within acceptable limits. Under typical operating conditions, the JS-based EMS successfully achieves system objectives. The DS for this deterministic approach is 100%, indicating that the requested power has been met. Although this approach performs well under normal conditions, if a disturbance occurs, it will be unable to account for it, resulting in a suboptimal solution.

**Fig. 14:**
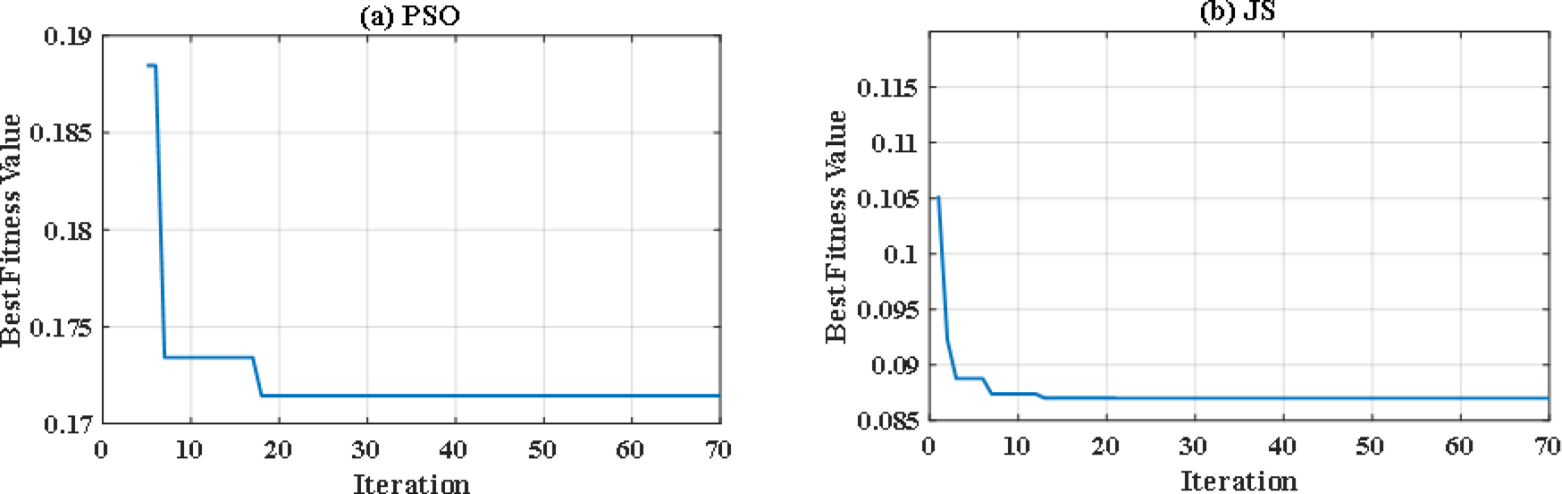
Convergence curve for **(a)** PSO and **(b)** JS.

**Fig. 15:**
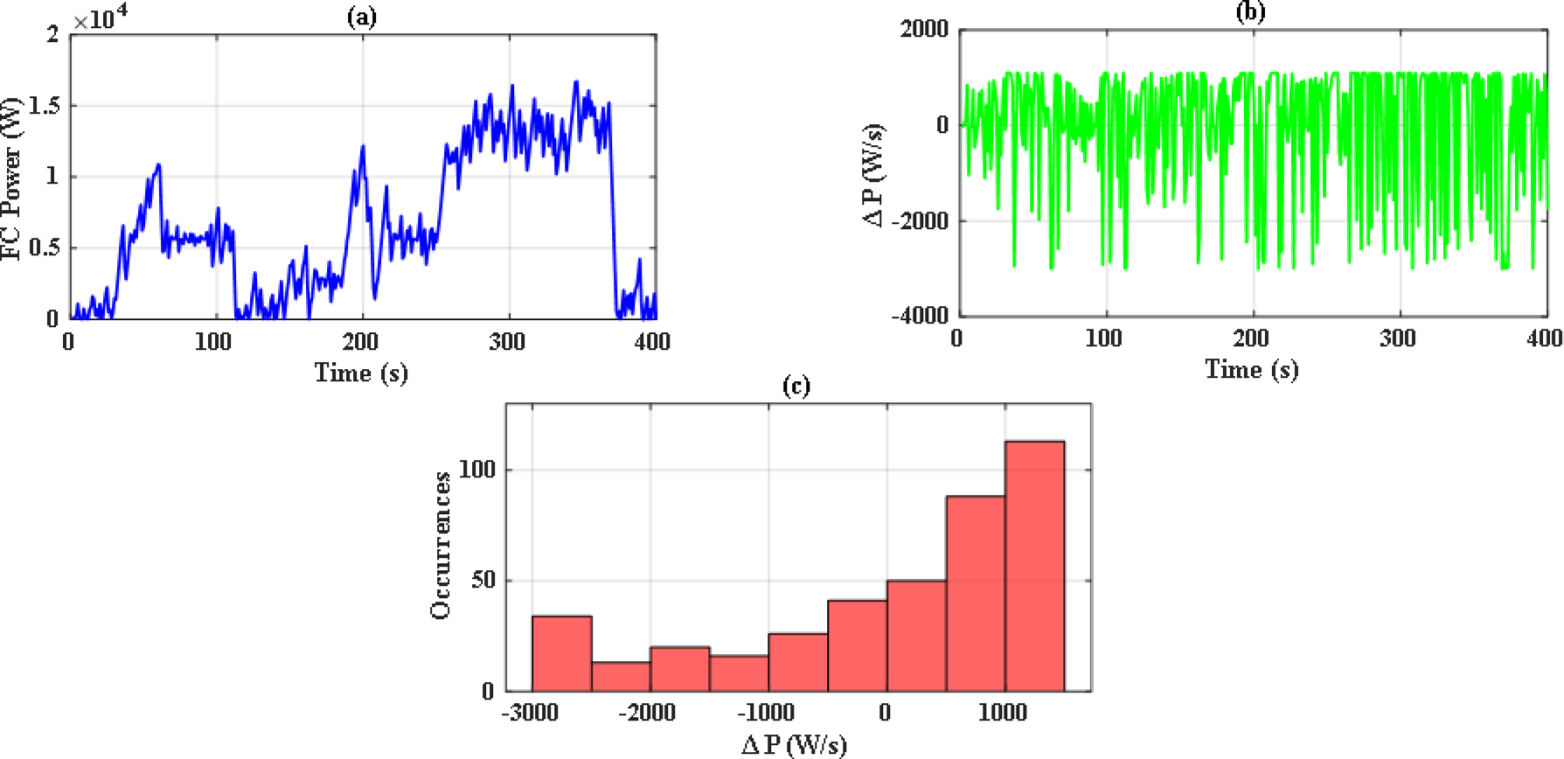
Stress analysis for PSO with EUDC.

**Fig. 16:**
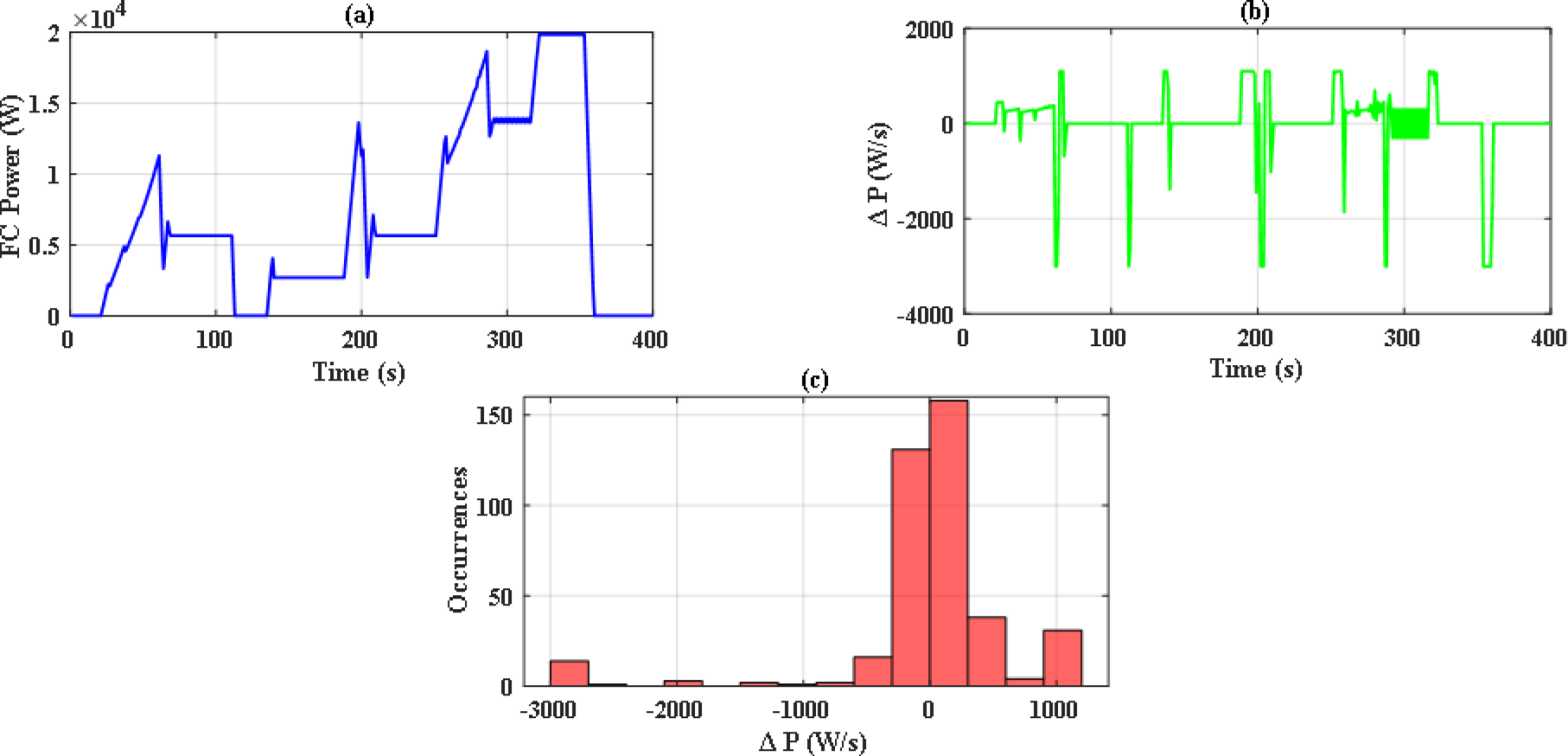
Stress analysis for JS with EUDC.

**Fig. 17:**
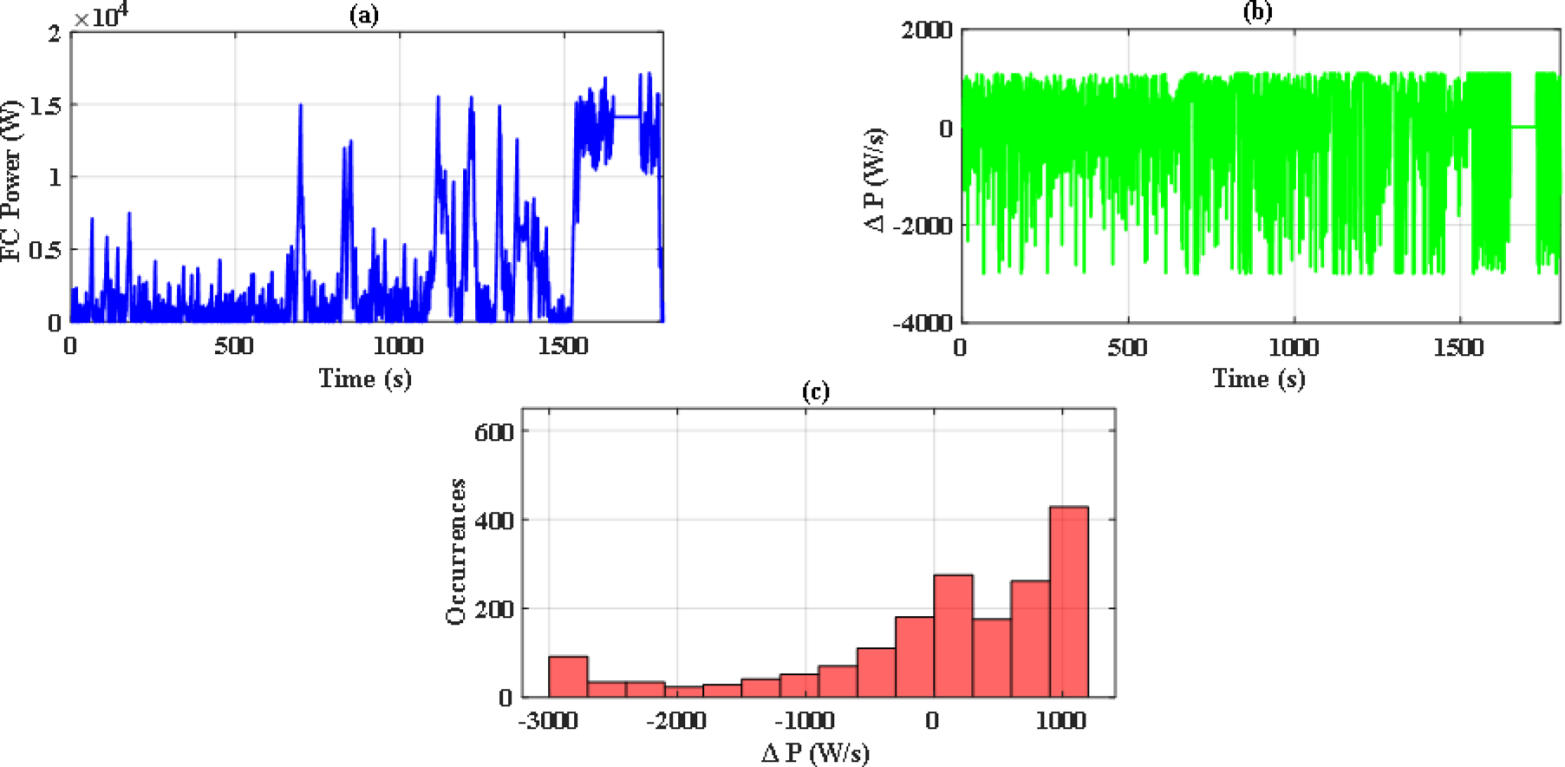
Stress analysis for JS with WLTC-Class 2.

**Fig. 18:**
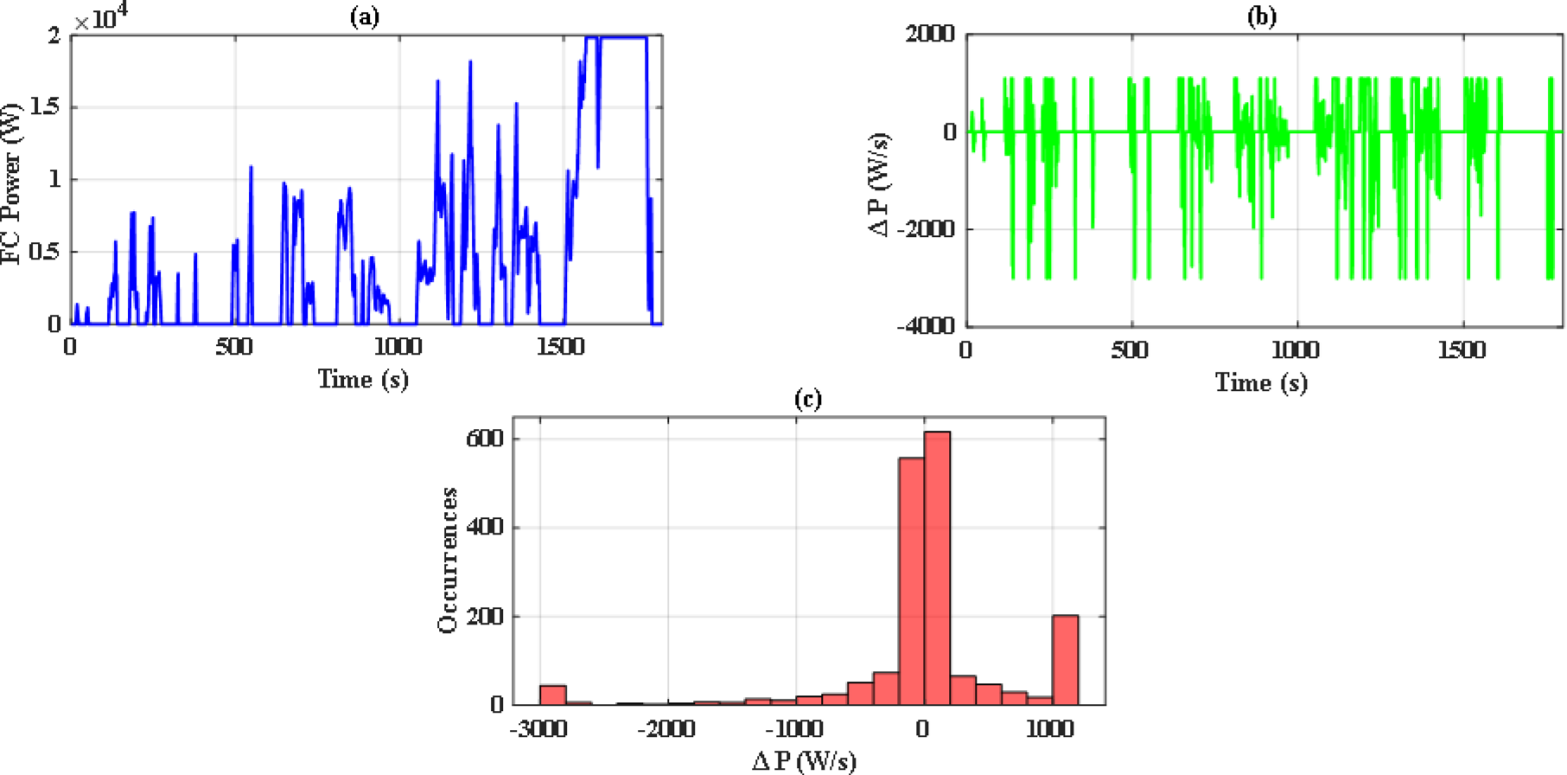
Stress analysis for JS with WLTC-Class 2.

So, with iterations number kept to 70, there were significant differences between the performance of both optimization methods. Figure 14(a) and Fig. 14(b) illustrate the convergence curve of PSO and JS, respectively. As noted, JS provides a much lower value for the cost function. Also, since there are severe changes in power demand at sudden hill climbing, rapid deceleration, and immediate acceleration, the EMS must manage to distribute power without stressing the FC. The power supplied by the FC in each case has been analyzed as in Figs. 15, 16 and 17, and Fig. 18. Although both techniques have been able to keep the rate of change of fuel cells power (Δ P) within the limits, the severe rate of power change occurs much more in the case of PSO, as the histograms show. On the other hand, the JS provides a more stable change of power, which reduces the stress on the FC, hence increasing the lifetime of the system. Moreover, by comparing the computational time of both methods as in Table 4, JS takes less time, even with the same iteration number. From the UC point of view, JS manages to reduce the stress on it by reducing the depth of discharge (DOD), which could effectively increase its lifetime. All these metrics are along with having a DS of 100% for both drive cycles with JS, in opposite to PSO, which has only 90.8% for WLTC-Class 2 as indicated in Table 5.

**Table 4:** Computing time required for each optimization method.

Solution techniques	Computation time	
	EUDC	WLTC-Class 2
PSO	0.1588	0.1629
JS	0.1501	0.1520

**Table 5:** Demand satisfaction indicator for each optimization method.

Solution techniques	Demand satisfaction indicator	
	EUDC	WLTC-Class 2
PSO	100%	90.8%
JS	100%	100%

### Robust optimization approach

**Fig. 19:**
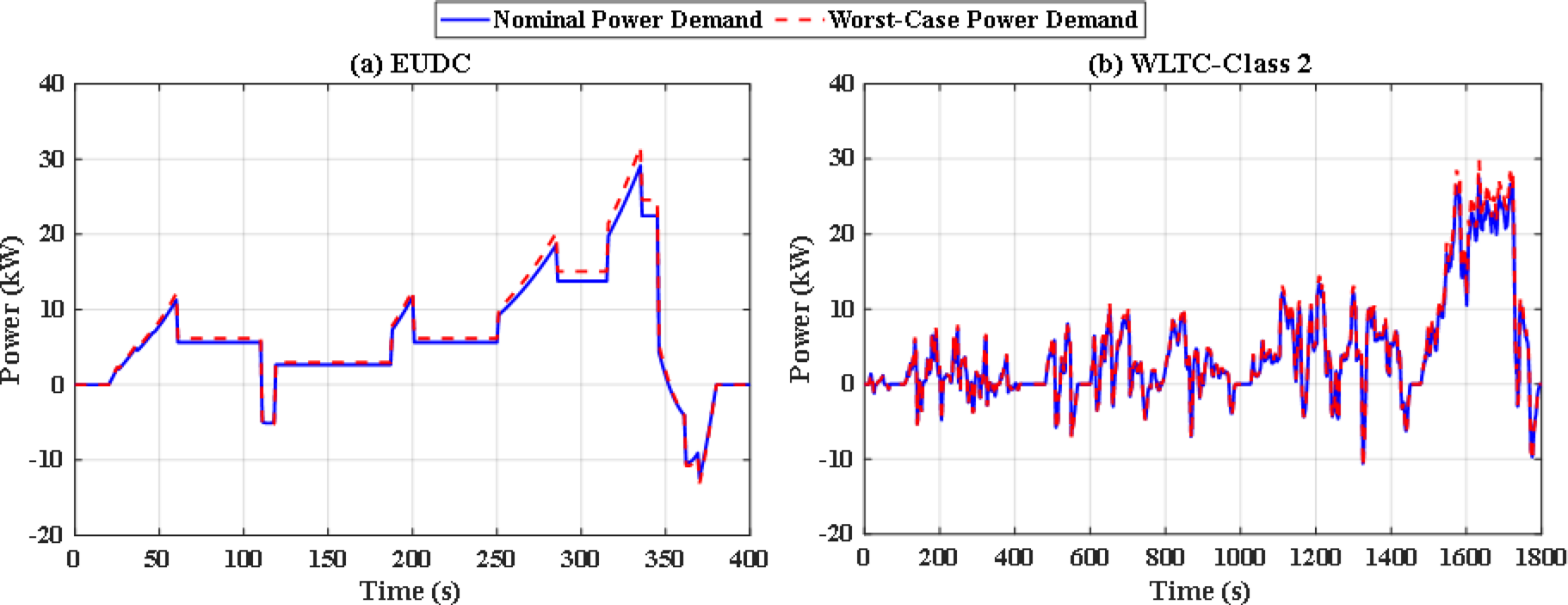
Nominal and worst-case power consumption for **(a)** EUDC and **(b)** WLTC-Class 2.

All the uncertainties are included for the entire simulation duration. By taking the uncertainty parameters, the worst-case demand power certainly differs from the nominal demand power. The nominal and perturbed demand power for EUDC and WLTC-Class 2 driving cycles are shown in Fig. 19(a) and Fig. 19(b) respectively.

**Fig. 20:**
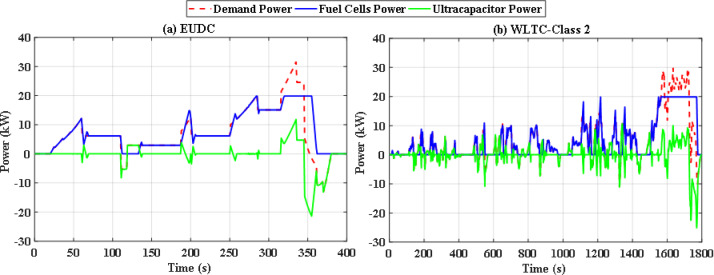
Robust JS-based optimization power distribution for **(a)** EUDC and **(b)** WLTC-Class 2.

**Fig. 21:**
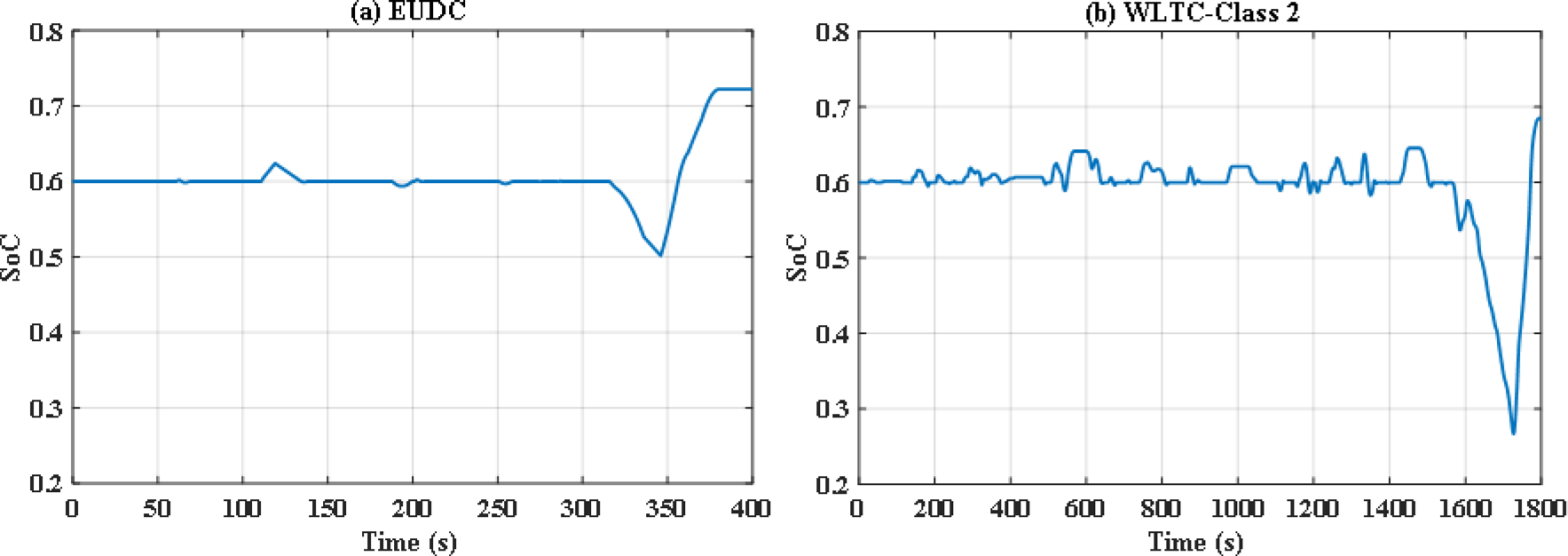
Robust JS-based optimization SoC of the ultracapacitor for **(a)** EUDC and **(b)** WLTC-Class 2.

**Fig. 22:**
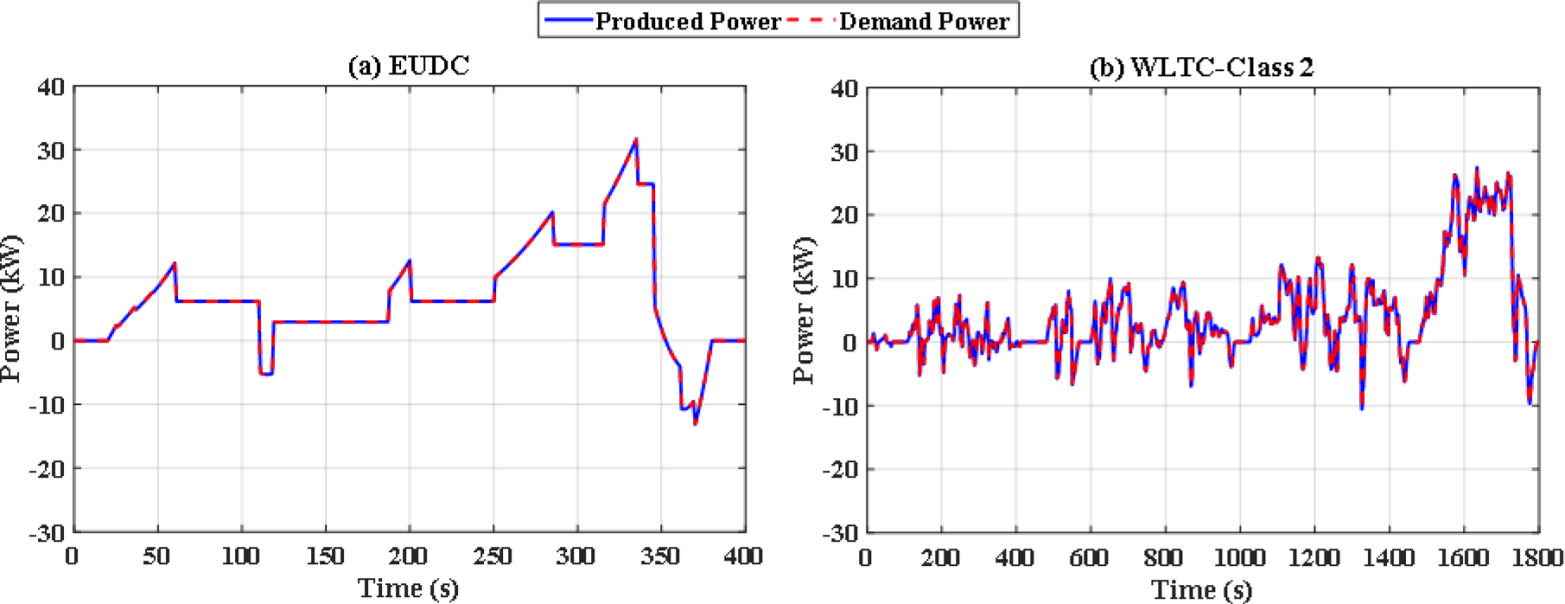
Robust JS-based optimization demand and produced powers for **(a)** EUDC and **(b)** WLTC-Class 2.

Without doubt the perturbed power demand exceeds those nominal values, so robust JS based algorithm is applied. Figure 20(a) and Fig. 20(b) show instantaneous power distribution among different sources for EUDC and WLTC-Class 2 driving cycles respectively, while Fig. 21(a) and Fig. 21(b) display instantaneous state of charge of the UC module. Figure 22(a) and Fig. 22(b) show the difference between the demand and produced power for both EUDC and WLTC-Class 2. As seen in the results, the robust JS based is able of providing the required power all over the duration of both driving cycles despite the existence of the six uncertainties, so the DS for this case is also 100%. The SoC is kept within the permissible range even with this worst case. This means that this algorithm can satisfy the system constraints and provide good performance for the vehicle.

Table 6 demonstrates the worst-case demand power $\:{(\text{P}}_{\text{d},\text{w}\text{c}})$ and power produced, consumed in deterministic and robust cases ($\:{\text{P}}_{\text{p},\text{d}}$ & $\:{\text{P}}_{\text{p},\text{r}}$). Using the perturbed power case data, the robust algorithm’s DS is 100% in both driving cycles, whereas the deterministic algorithm’s DS in EUDC and WLTC-Class2 is only 91.35% and 91.34%, respectively. This power shortfall may cause the vehicle to decelerate and have mediocre performance. However, this resilience of the robust approach will come at a price, as the CR for EUDC and WLTC-Class2 are 10.8% and 11.17% increase in hydrogen consumption.

**Table 6:** Consumed power (kW).

Driving cycle	$\:{\text{P}}_{\text{d},\text{w}\text{c}}$	$\:{\text{P}}_{\text{p},\text{d}}$	$\:{\text{P}}_{\text{p},\text{r}}$	$\:{\text{P}}_{\text{d},\text{w}\text{c}}-{\text{P}}_{\text{p},\text{d}}$
EUDC	2731.1	2495	2731.1	236.1
WLTC-Class 2	8214.4	7503.6	8214.4	710.8

## Conclusions

This paper introduces an optimization-based EMS for an FCHEV, including fuel cells and ultracapacitors. Firstly, JS optimizer was adopted for this problem, and its results were compared to the results obtained by PSO, which is a commonly used optimization technique. Under a limited number of iterations, PSO struggles to fulfill the demanded power of the vehicles in some cases due to infeasible solutions. On the other hand, JS optimizer outperforms the PSO by providing optimal solutions while meeting all the system operational constraints under the same conditions. By performing stress analysis of the power of fuel cells, it was found that JS has fewer occurrences of severe changes (1.1 kW/s and − 3 kW/s) in the power. Moreover, it was able to reduce the computational time by 5.524% and 6.685% for EUDC and WLTS Class-2, respectively, with respect to that of PSO. Also, the depth of discharge was 46.87% and 58.76% for EUDC and WLTS Class-2. This indicates that JS can operate successfully for energy management under various drive cycles with acceptable computational time.

Due to the uncertainties that may occur during the driving cycle, a deterministic algorithm may fail to provide the power demand with DS 91.35% and 91.34% in EUDC and WLTS Class-2, respectively. To counteract that problem, robust optimization (RO), including the min-max approach with JS, was employed. The performance of the RO-based EMS was evaluated under the worst-case conditions and compared with the deterministic framework using JS. It was shown that, despite the uncertainties that may occur, the RO-based EMS provides all system requirements with DS 100% as opposed to the what-happens deterministic framework JS. However, this robustness comes at a cost. The cost of robustness (CR) for the dive cycles was found to be 10.8% and 11.17% increase in hydrogen consumption. This higher cost in hydrogen consumption ensures that the system can cope with uncertainties. This increase in cost can be considered as a trade-off.

## Data Availability

All data generated or analyzed during this study are included in this published article.
